# Strigolactones: new plant hormones in action

**DOI:** 10.1007/s00425-015-2455-5

**Published:** 2016-02-02

**Authors:** Binne Zwanenburg, Tomáš Pospíšil, Sanja Ćavar Zeljković

**Affiliations:** Department of Organic Chemistry, Institute for Molecules and Materials, Radboud University Nijmegen, Heyendaalseweg 135, 6525 AJ Nijmegen, The Netherlands; Department of Growth Regulators, Faculty of Science, Centre of Region Haná for Biotechnological and Agricultural Research, Palacky University, Slechtitelu 27, 78371 Olomouc, Czech Republic; Central Laboratories and Research Support, Faculty of Science, Centre of Region Haná for Biotechnological and Agricultural Research, Palacky University, Slechtitelu 27, 78371 Olomouc, Czech Republic

**Keywords:** Karrikins, Mode of action, Signal transduction, Strigolactones, Strigolactone analogs, Strigolactone mimics

## Abstract

*****Main conclusion***:**

**The key step in the mode of action of strigolactones is the enzymatic detachment of the D-ring. The thus formed hydroxy butenolide induces conformational changes of the receptor pocket which trigger a cascade of reactions in the signal transduction.**

**Abstract:**

Strigolactones (SLs) constitute a new class of plant hormones which are of increasing importance in plant science. For the last 60 years, they have been known as germination stimulants for parasitic plants. Recently, several new bio-properties of SLs have been discovered such as the branching factor for arbuscular mycorrhizal fungi, regulation of plant architecture (inhibition of bud outgrowth and of shoot branching) and the response to abiotic factors, etc. To broaden horizons and encourage new ideas for identifying and synthesising new and structurally simple SLs, this review is focused on molecular aspects of this new class of plant hormones. Special attention has been given to structural features, the mode of action of these phytohormones in various biological actions, the design of SL analogs and their applications.

## Introduction

Strigolactones (SLs) constitute a new class of plant hormones which are of increasing importance in plant science. They belong to the group of biologically active molecules called semiochemicals that are used to disseminate information between individual species. Important examples of plants that have become completely dependent on allelochemicals are the parasitic weeds witchweed (*Striga* spp., Orobanchaceae/Scrophulariaceae) and broomrape (*Orobanche* spp., Orobanchaceae). The seeds of these weeds only germinate in response to specific chemicals, namely germination stimulants, present in the rhizosphere of host plants and some non-host plants. For these parasitic flowering plants, which are totally dependent on specific association with a host that provides nutrients and water, this system ensures that germination only starts when suitable host roots are available in the immediate vicinity. Other allelochemicals are required to effect attachment of the germinated seeds to the roots of the host plants via a specialised organ, the *haustorium*. Once the vascular connections between host and parasite have been established, the parasite can develop at the expense of the host plant. As a consequence of providing nutrients to the parasite, the crop yield of the host plant will be severely affected. In many cases of important food crops, this parasitic interaction causes a serious problem in food production.

In recent years several new bio-properties of SLs have been discovered. A real breakthrough was the discovery that SLs act as the branching factor for arbuscular mycorrhizal (AM) fungi (Akiyama et al. 2005; Parniske 2008). Mycorrhizae are symbiotic associations between soil, fungi and plant roots (Akiyama and Hayashi 2006). This interaction is probably the most widespread and significant symbiosis in nature (Brachmann and Parniske 2006). AM fungi are obligate symbionts unable to complete their life cycle in the absence of a suitable host. A critical step in the development of AM fungi is the triggering of the hyphal morphogenesis by a branching factor. The isolation and characterization of a branching factor was extremely difficult due to the fact that their concentrations were very low. The first branching factor was isolated from the roots of hydroponically grown *Lotus japonica* and it was shown to be (+)-5-deoxystrigol. It was also demonstrated that other SLs, such as strigol and orobanchol, are highly active branching factors. Knowing the identity of the branching factors of AM fungi opens new windows for their practical applications (Akiyama and Hayashi 2006).

A second important breakthrough in SL research followed a few years later. It was then demonstrated that endogenous SLs play an important role in the control of plant architecture. Inhibition of bud outgrowth and inhibition of shoot branching are typical examples (Gomez-Roldan et al. 2008; Umehara et al. 2008). The inhibitory processes are regulated by endogenous cues of which SLs are probably most prominent. Importantly, inhibition of shoot branching could also be induced exogenously by treatment with the synthetic SL GR24. For a long time, involvement of two other classes of plant hormones, namely auxin and cytokinines, has been known in controlling shoot branching. Now, SLs are recognised as a third class of new plant hormones. This control of plant architecture with SLs gave rise to an avalanche of publications on this topic, indicating the high importance of this new role of SLs. Several excellent reviews have appeared on this subject (Tsuchiya and McCourt 2009; Koltai 2011, 2014, 2015; Cheng et al. 2013; Waldie et al. 2014).

In this review, the focus will primarily be on **molecular** aspects of this intriguing class of new plant hormones. The synthesis of SLs, of both naturally occurring SLs and of synthetic analogs, are reviewed separately (Zwanenburg et al. 2015).

## Isolation of SLs

The first SL ever isolated was obtained from root exudates of cotton (*Gossypium hirsutum* L.) as early as 1966 and was named strigol (Cook et al. 1966). The gross structure of strigol was elucidated in 1972 (Cook et al. 1972) and the full details were determined by means of an X-ray diffraction analysis in 1985 (Brooks et al. 1985) about 20 years after its isolation. Strigol was isolated from a non-host for the parasitic weed *Striga* and consequently, its significance for the host–parasite interaction was uncertain for a long time. It was not until 1992 that sorgolactone, a compound with a structure similar to strigol, was isolated (Hauck et al. 1992) from root exudates of a true host for *Striga*, sorghum (*Sorghum bicolor* L. Moench).

Soon thereafter, alectrol was obtained from the root exudate of cowpea (*Vigna unguiculata* L.) which is a host for *S. gesnerioides* (Muller et al. 1992). The collective name ‘strigolactones’ was proposed by Butler, a pioneer in this area (Butler 1995). The isolation of SLs from root exudates is very laborious and requires a careful chromatographic separation accompanied by bioassays for germination of appropriate seeds of parasitic weeds. The production of SLs per plant is very small: 15 pg/day/plant (Sato et al. 2005), hence collection of root exudate from hydroponically grown host plants requires an experimental set-up with many plants. At present the HPLC separation techniques are much more sophisticated and fewer plants are needed. The structural analysis of SLs is a highly demanding exercise using high resolution mass spectrometry and NMR analysis. Especially, establishing the correct stereochemistry needs utmost care.

**Fig. 1:**
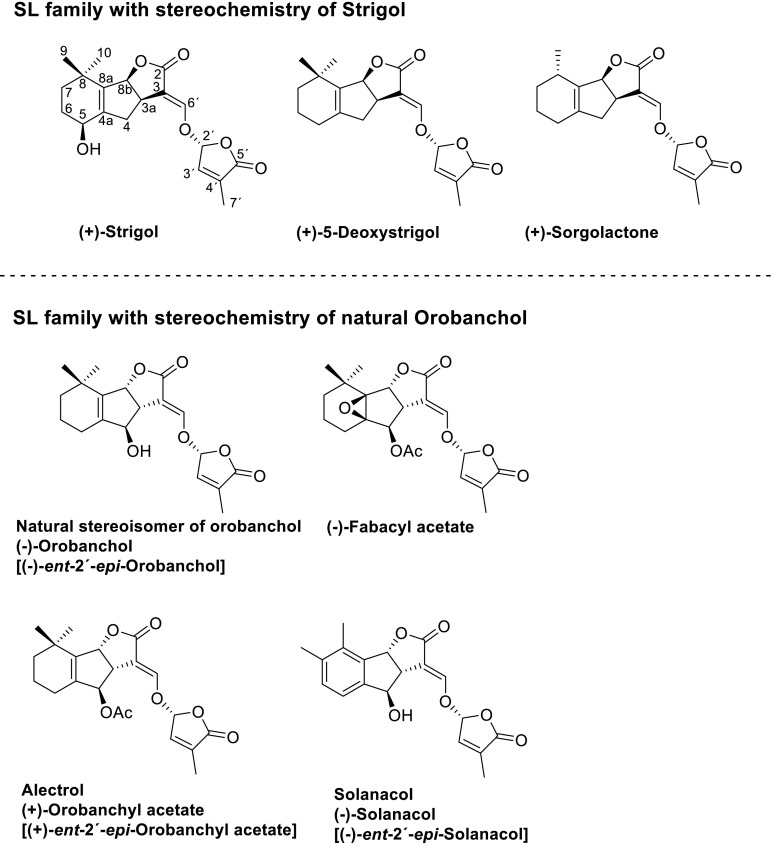
Structures of naturally occurring SLs. Names of the orobanchol family using (+)-strigol as the parent compound are given within *brackets*

SLs invariably contain three annelated rings, the ABC scaffold, connected by means of an enol ether unit with a butenolide ring, the D-ring (Fig. 1).

## Naturally occurring SLs

At present two families of naturally occurring SLs are known (Fig. 1). Because of the tricky aspects of the structural analyses, some misassignments were made. For example, establishing the structure of alectrol (Muller et al. 1992) was particularly difficult and it took about two decades before the correct structure was elucidated (Ueno et al. 2011, 2015). The structure of orobanchol, which is probably one of the most abundant SLs, was initially incorrectly assigned (Ueno et al. 2011). Originally it was a logical assumption that the stereochemistry would be as in (+)-strigol (Mori et al. 1999). A third example is solanacol. In the first proposed structure the methyl substituents in the A-ring were positioned *para* (Xie et al. 2007) instead of *ortho* (Takikawa et al. 2009), and as far as the stereochemistry is concerned: it belongs to the orobanchol family and not to the strigol family as suggested originally (Chen et al. 2010, 2013). More details about the structural corrections have been reviewed earlier (Zwanenburg and Pospisil 2013).

The occurrence of SLs in nature and the source from where they have been obtained has recently been reviewed and where possible correct structures are included in the tables (Cavar et al. 2015). Moreover, strigolactones play a major role in host specificity of *Orobanche* and *Phelipanche* (the broomrapes) seed germination. In general, weedy broomrape species are less specialised in germination requirements than the non-weedy species (Fernandez-Aparicio et al. 2011).

## Relevance of stereochemistry in SLs

Establishing the stereochemistry at the respective stereogenic centers was, and still is, a major obstacle in elucidating the correct detailed structure of naturally occurring SLs. For assigning the stereochemistry at C-2′ of the D-ring the empirical rule reported by Welzel et al. (1999), based on the Cotton effect in ORD/CD spectra, is appropriate. For the ABC part, correlation diagrams with compounds of known stereochemistry are mostly used (Zwanenburg and Pospisil 2013). An X-ray diffraction analysis is the most reliable manner to establish the absolute stereochemistry of an SL. However, for that a crystalline sample of the SL is needed which is not always easy to obtain. The stereochemistry has a pronounced effect on the germinating activity towards the seed of parasitic weeds. In addition, for the other SL bio-properties there is a profound effect of the stereochemistry on the bio-response.

## Naming protocol for SLs

The SLs have several chiral centers, for example strigol has three such centers and there are 2^3^ = 8 conceivable stereoisomers. From a chemical point of view a correct and unambiguous manner to designate the chirality at the respective stereogenic centers, the use of the Cahn-Ingold-Prelog (CIP) descriptors *R* and *S* to indicate the sense of chirality is most appropriate. The *R*,*S* notation is based on abstract rules which are not easy to handle. Using the *ent* and *epi* prefixes is much easier in practise, whereby *ent* refers to enantiomer, i.e. mirror image of an entire unit and *epi* refers to epimer, i.e. opposite configuration at a given atom. For the *ent*/*epi* method it is necessary to choose a reference compound, a parent molecule. In the time before the structural correction of orobanchol, the naming of SLs was simple and straightforward: (+)-strigol was the logical parent compound and the stereochemistry of all other SLs was related to that parent compound. However, after the structure change of orobanchol in 2011 (Ueno et al. 2011) there were two options, either to keep the naming protocol with (+)- strigol as the parent or to use the new structure for natural orobanchol as parent compound for the orobanchol family. Both methods are in use, which may lead to confusing situations (Zwanenburg and Pospisil 2013). The reader is forewarned.

Scaffidi et al. (2014) suggested an alternative naming and notation in the structural correlation of GR24 stereoisomers using both (+)-strigol and (−)-orobanchol as standards. This resulted in two names for some stereoisomers, e.g. *ent*-2′-*epi*-5-deoxystrigol is also named 4-deoxyorobanchol. This method has little added value and is confusing for those who are less familiar with stereochemical issues.

## Simplified SLs with retention of germinating activity: design of SL analogs

**Fig. 2:**
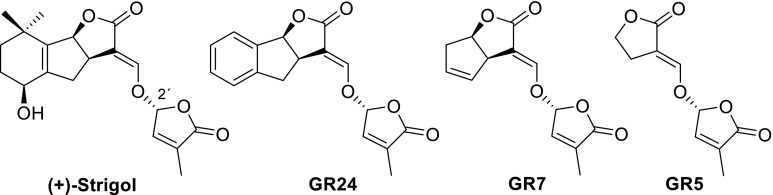
Simplification of SL structures (all are appreciably active as germinating agents)

**Fig. 3:**
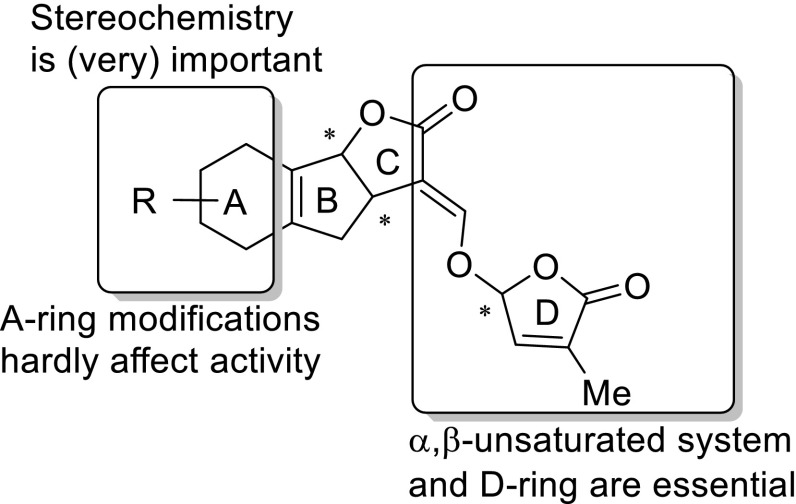
Model for designing SL analogs with germinating activity

**Fig. 4:**
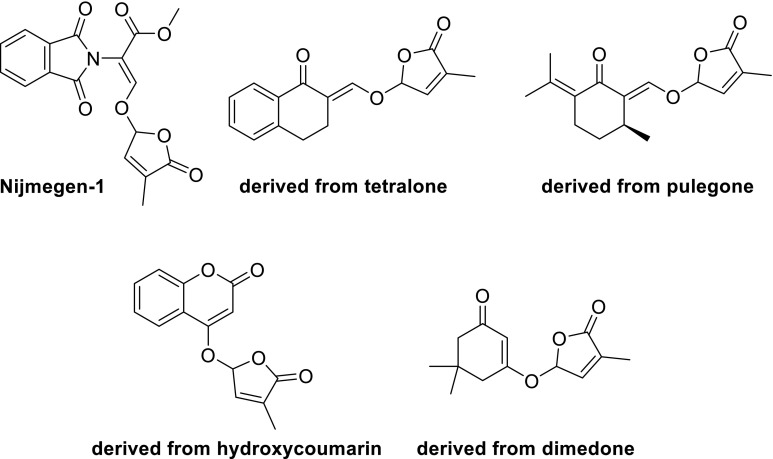
Synthetic analogs of SLs with germinating activity

Naturally occurring SLs have a too complex structure for synthesis on a multi-gramme scale (Zwanenburg et al. 2015). The total synthesis of several natural SLs has been accomplished, but linear sequences of many steps, >20 or more, were required. To study the effect of SLs on various biological processes, model compounds were designed and prepared. A prerequisite is that these SL analogs have a (much) simpler structure than natural SLs, but that their bio-activity is largely retained. For a rational design of SL analogs, it is necessary to identify the bioactiphore, i.e. that part of the molecule that is primarily responsible for bioactivity. To this end the structure of a natural SL, say strigol, is systematically simplified. Making the A-ring aromatic leads to a compound which is code named GR24 after its inventor Gerald Rosebery, removal of the A-ring gives GR7 and cutting of the B-ring leads to GR5. All these GR compounds are appreciably active as germination stimulant for parasitic weeds (Fig. 2). However, when the C-ring is removed the activity is lost. This implies that the bioactiphore resides in the CD part of SLs. The information presented above allows the design of a model compound for SL analogs with germinating activity (Fig. 3). A typical feature of the model is that there is a considerable molecular freedom in the A-ring part of the molecule. Stereochemistry is important as mentioned in the preceding section. This model has been used successfully to design a large series of highly active SL analogs. Some typical examples are shown in Fig. 4.

It is important to note that these analogs not only must have a simplified structure with retention of germinating activity, but also they must be synthetically readily accessible. An illustrative example of the successful implementation of the model is Nijmegen-1. It can indeed readily be obtained from simple starting materials in a few synthetic steps and its germinating activity is comparable to that of GR24.

**Fig. 5:**
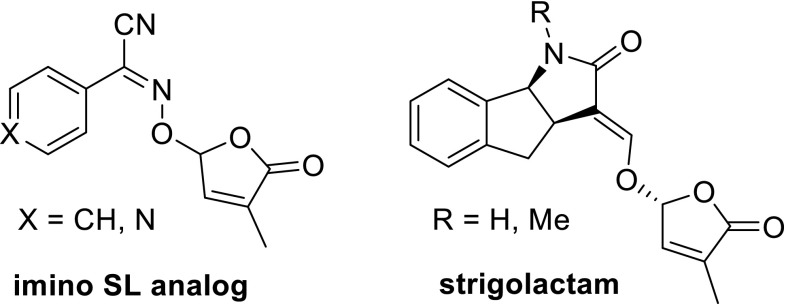
Isosteric SL analogs

An alternative way of designing SL analogs with germination capabilities is isosteric replacement of a particular atom; in the case of SLs, most logically an oxygen atom is replaced by another heteroatom. There are two successful examples of such an isosteric replacement, namely: imino SL analogs (Kondo et al. 2007) and strigolactams (Lachia et al. 2015) (Fig. 5). In the imino analogs the electron-withdrawing CN is essential for activity.

GR24 is commonly used as standard in germination studies. Mostly, this stimulant is a racemate in which the relative stereo configuration is as in (+)-strigol. However, it should be noted that not all seeds of parasitic weeds do respond to GR24, for example *O. crenata*, *O. foetida*, *O. hederae* and *O. densiflora* (Fernandez-Aparicio et al. 2011), as well as *O. picridis* and *O. minor* subsp. *maritima* (Thorogood et al. 2009) do not respond.

## SL mimics

**Fig. 6:**
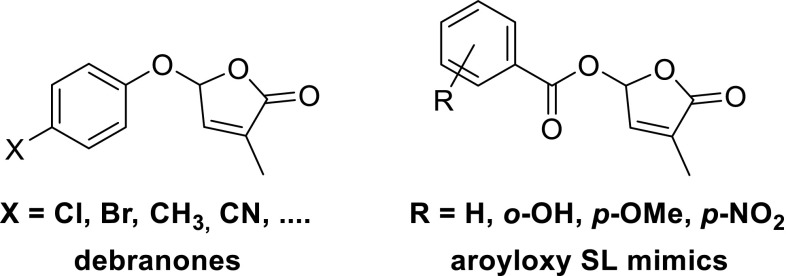
Debranones and aroyloxy SL mimics

An interesting and unexpected development is that a group of compounds lacking the ABC scaffold also can stimulate germination. These compounds are named as SL mimics, as they mimic the SL activity but do not have the typical SL structural features: a D-ring connected with an α,β-unsaturated carbonyl via an enol ether unit. One group of substituted D-ring compounds with germinating activity has an aryloxy substituent at C-5 (Fig. 6). These compounds were named as debranones (branching furanones) because the main activity profile is inhibition of shoot branching (Fukui et al. 2011, 2013). Seeds of *Striga hermonthica* respond modestly to these debranones. It was found that *para*-chlorophenoxy-debranone had the highest activity. So far *Orobanche* seeds were not tested with debranones. The second group of SL mimics that was discovered almost at the same time has an aroyloxy substituent at C-5 of the D-ring (Zwanenburg et al. 2011, 2013). These SL mimics are moderately active as germination stimulant towards seeds of *S. hermonthica* but remarkably active in the case seeds of *Orobanche cernua* and *Pelipanche ramosa* seeds. A remarkable finding was that introduction of an extra methyl group at C-4 gave SL mimics which were inactive as germination stimulant. This structural change in SL mimics may give a clue for their mode of action. So far the inhibition of shoot branching of these aroyloxy SL mimics has not been tested, but experiments to end this are ongoing. The area of SL mimics clearly still in its infancy.

## SLs as branching factors for AM fungi

**Fig. 7:**
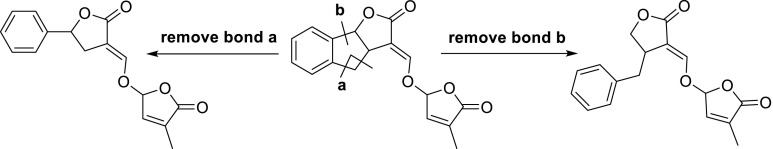
Simplified SL structures with activity as a branching factor for AM fungi

A new and important discovery is the role of SLs as branching factors for arbuscular mycorrhizal (AM) fungi (Akiyama et al. 2005; Parniske 2005, 2008). The structure–activity relationship of SLs as branching factors was extensively studied by Akiyama et al. (2010), see also Besserer et al. (2006). It was found that (+)-orobanchol had the highest activity followed by 5-deoxystrigol. (+)-GR24 is very active, almost as active as (+)-strigol, but its mirror image practically is not (10,000 times less active). GR7 which is lacking the A-ring is 1000 times less active than GR24 whilst *ent* GR7 is almost inactive. This information suggests that for an SL analog to be active as a branching factor for AM fungi all rings of the ABC scaffold need to be there and also that the stereochemistry must be as in the strigol family. This implies that there is not much molecular freedom to design simpler structure for interaction with AM fungi. However, it was found recently that the B-ring is not strictly necessary. The SL analogs as shown in Fig. 7 where a phenyl is connected with the γ-carbon of the D-ring or where a benzyl group is attached to the β-position, both are appreciably active as branching factors (Akayama, personal communication, 2015). Both compounds are not difficult to prepare as it had been reported previously (Nefkens et al. 1997).

Stimulation of AM fungi fulfils a symbiotic role with parasitic plants. After the first observation, much attention was given to the beneficial mutualistic and symbiotic associations of AM fungi and parasitic plants (Akiyama and Hayashi 2006; Bonfante and Requena 2011). AM fungi facilitate the uptake of phosphates and nitrates, and in a sense these fungi serve as soil fertiliser which may be of agricultural value. Knowledge of this symbiotic relationship could provide a new strategy for the management and control of beneficial fungal symbionts and of devastating parasitic weeds in agriculture and natural ecosystems.

## SLs as inhibitors for shoot branching and in their role in controlling plant architecture

As mentioned in the introduction, SLs are now recognised as new plant hormones. An important newly discovered activity deals with the control of plant architecture. SLs will not operate standing alone, but in concert with other plant hormones. Until 25 years ago there were 5 types of plant hormones known, namely: auxins, cytokinins, ethene (ethylene), gibberellins and abscisic acid (ABA). More recently, brassinosteroids and jasmonates have been added to the list. The role of the various plant hormones in the plant kingdom is under extensive investigation. There is accumulating evidence that SLs interplay in a crosstalk with several of these plant hormones. Which endogenously SLs are operative in the interplay *in planta* is unknown in most cases. The crosstalk of SLs with other plant hormones may either take place in a fully concerted manner or sequentially in a cascade of events, although in many cases the precise *modus operandi* is not known in detail. Phenomenologically, the crosstalk interactions are well documented.

As it is common for other phytohormones, the SL biosynthesis and activity is regulated by other hormones. For instance, cytokinins act as antagonists to SLs in regulation of axillary bud outgrowth (Dun et al. 2012) and in regulation of mesocotyl elongation in darkness (Hu et al. 2014). Auxins are not only shown as one of the major regulators of SL biosynthesis (Hayward et al. 2009; Al-Babili and Bouwmeester 2015, and references therein), but also they act as antagonists because SLs may enhance auxin transport (Cheng et al. 2013, and references therein). Lopez-Raez et al. (2010) showed that abscisic acid, one of the key regulators of plant response to abiotic stress, has a role in SL biosynthesis, but, on the other hand SLs can also impact biosynthesis of abscisic acid (Al-Babili and Bouwmeester 2015). Besides phytohormones, it is well established that phosphate affects SL biosynthesis, meaning that shortage of phosphate increases SL production (Koltai 2015, and references therein).

However, all these facts are still on cellular level, and they do not explain on a molecular basis which exact mechanisms play a role. This is a highly complex research area due to the different effects of phytohormones and varying context of their actions.

Most studies on the control of plant architecture are carried out with increased branching mutants, predominantly with *ramosus* (*rms*) in garden pea (*Pisum sativum*), *more axillary growth* (*max*) in Arabidopsis (*Arabidopsis thaliana*), *decreased apical dominance* (*dad*) in *Petuniahybrida* and *dwarf* (*d*) and hig*h tillering dwarf* (*htd*) in rice (*Oryza sativa*). Treatment with an exogenous SL, practically in all cases synthetic GR24 was employed, resulted in the inhibition of shoot branching (Dun et al. 2013), stimulation of internode growth (de Saint et al. 2013), acceleration of leaf senescence (Yamada et al. 2014), enhance root hair elongation and the growth of primary roots (Kapulnik et al. 2011), inhibition of the outgrowth of axillary buds (Minakuchi et al. 2010), inhibition of formation of adventitious and lateral roots (Rasmussen et al. 2012a, b, 2013a, b), increasing stem thickness and inducing secondary growth (Agusti et al. 2011) and other morphological changes. It was found that auxin–SL interactions at multiple levels are critical for branching control (Stirnberg et al. 2010; Koltai et al. 2010). How these inhibitory processes work on a molecular level is still unknown. The plant physiology and biology of the control plant architecture induced by SLs are beyond the scope of this review. The relevant details of these aspect of the control of plant architecture on the cellular level are summarised in several excellent reviews (Tsuchiya and McCourt 2009; Koltai 2011, 2014, 2015; Cheng et al. 2013; Waldie et al. 2014).

**Fig. 8:**
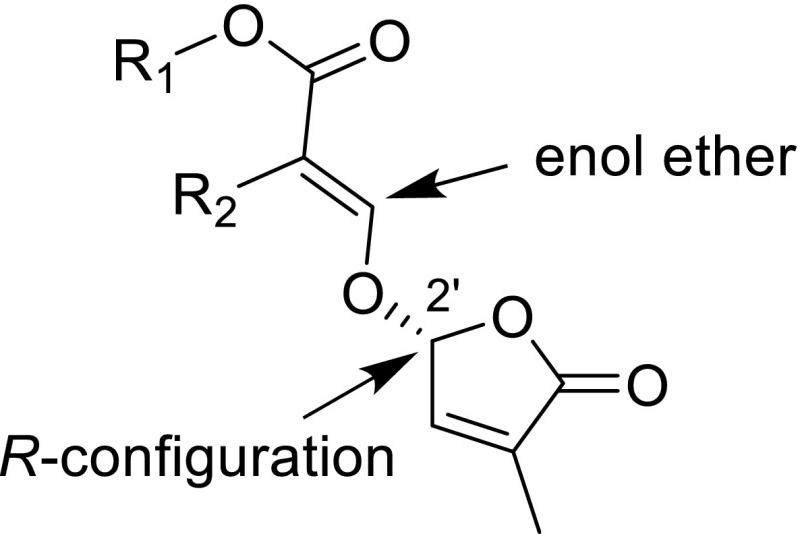
Minimum structure required for shoot branching inhibition in rice

The structural requirements are highly relevant for shoot branching inhibition. The inhibitory effect of a series of 30 compounds, including the naturally occurring SLs 5-deoxystrigol and orobanchol, and the synthetic SL analogs GR24, GR7 and GR5, was investigated with SL-deficient rice mutant *d10*. Some of these compounds were also studied for the effect on Arabidopsis mutant *max4* (Umehara et al. 2015). This structure–activity study revealed that the *R*-configuration at C-2′ of the D-ring in SLs is critical for hormonal activity in rice tillering. This stereochemistry is present in practically all natural SLs. By truncation of the A- and B-ring of the natural SLs the minimum structure for activity, involving the D-ring, the enol ether moiety conjugated with ester unit (Fig. 8) was established. Essentially, the truncation method was the same as that used for the design of germination stimulants (Zwanenburg and Pospisil 2013). Hence, the design model shown in Fig. 3 may also be applicable for shoot branching inhibitors. This idea opens new avenues for identifying and synthesising new and structurally simple SL analogs for the control of plant architecture. Such compounds may be potential candidates for agricultural applications.

**Fig. 9:**
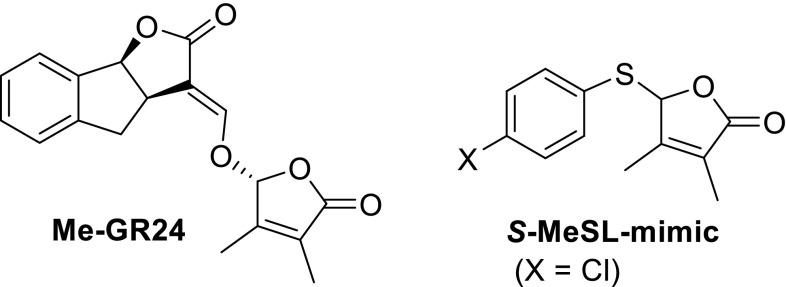
SLs with an extra methyl group at C-3′ in the D-ring

It is of interest to note that GR5, the AB-ring truncated analog, strongly inhibits shoot branching when applied hydroponically, whereas application to the axillary bud of Arabidopsis only gave a weak response. There is a profound difference between rice and pea when treated with a branching controlling inhibitor. In peas a structure–activity study for shoot branching, employing the SL-deficient mutant *rms1*, demonstrated that naturally occurring SLs, such as 5-deoxystrigol, strigol and orobanchol are all highly active but the stereochemistry at C-2′ is irrelevant, unlike in rice (Boyer et al. 2012, 2014). This was found for direct treatment of the axillary buds and in hydroponic culture system. Strigol and orobanchol have a lower response than the corresponding acetates probably due to the difference in lipophilicity. A remarkable observation was that an extra methyl group at C-3′ in GR24 has a boosting effect on the activity. Unexpectedly, an SL mimic having an S-aryl at C-2′ and an extra methyl group at C-3′ is surprisingly active (Fig. 9). It has not been made sure whether the aromatic group in this mimic is a prerequisite.

SL mimics (Fig. 9) with an inhibitory effect on shoot branching of rice mutants *d10*-*1* were reported by the Asami group (Fukui et al. 2011, 2013). All these mimics, which are also named as debranones (furanones showing de-branching activity), have O-aryl substituents at C-2′ of the butenolide ring. Mimics with a Br or a CN group in the *para* position are the most active ones. These compounds resemble the SL mimic reported by Boyer et al. having an S-atom at C-2′. Again, it is not made sure whether the O-aryl group is required for activity. Note that these debranones are also moderately active as germinating agents (see section SL mimics).

## SLs and karrikinolides (smoke compounds)

**Fig. 10:**
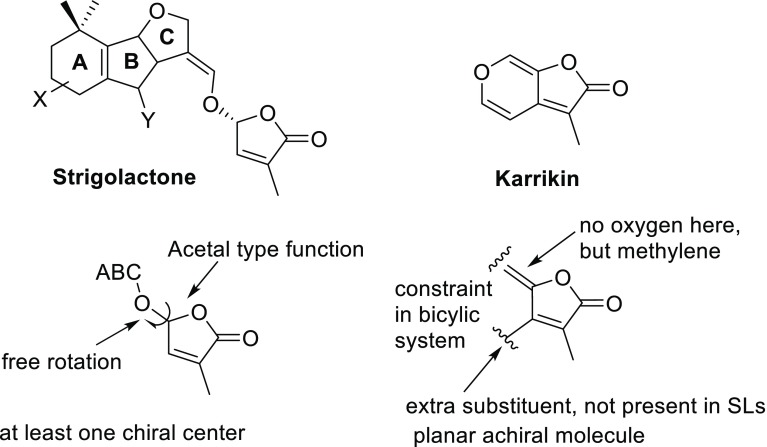
Comparison of structural features of SLs and KARs

An intriguing type of compounds was isolated from smoke of bush fires in Australia, which were named karrikins or karrikinolides (KARs), after the aboriginal word for smoke: ‘karik’ (Flematti et al. 2004; Waters et al. 2012). KARs contains a butenolide ring but its structure features differ profoundly from that of the butenolide in SLs (Fig. 10).

The KAR structure is planar and achiral, contains two annelated rings, whilst SLs have at least one chiral center, one of them at the five-membered D-ring. The five-membered ring in SLs can rotate freely while in KARs it is constraint in a rigid bicyclic system. The KARs contains an *exo*-methylene group at the γ-carbon of the lactone, while in SLs this is an acetal type group. It is evident, from the molecular point of view, that the compounds are quite different entities, each with its own reactivity pattern with practically no common features. In spite of this, KARs are germination stimulants for seeds of *Solanum orbiculatum*, but not for seeds of parasitic weeds (Flematti et al. 2010).

It is perfectly alright to discuss KARs in the same context as SLs because they both are germination stimulants, albeit for different seed types. However, the justification that is frequently encountered in the literature, namely that both stimulants contain a similar butenolide unit is simply not correct.

## Mode of action of SLs

**Fig. 11:**
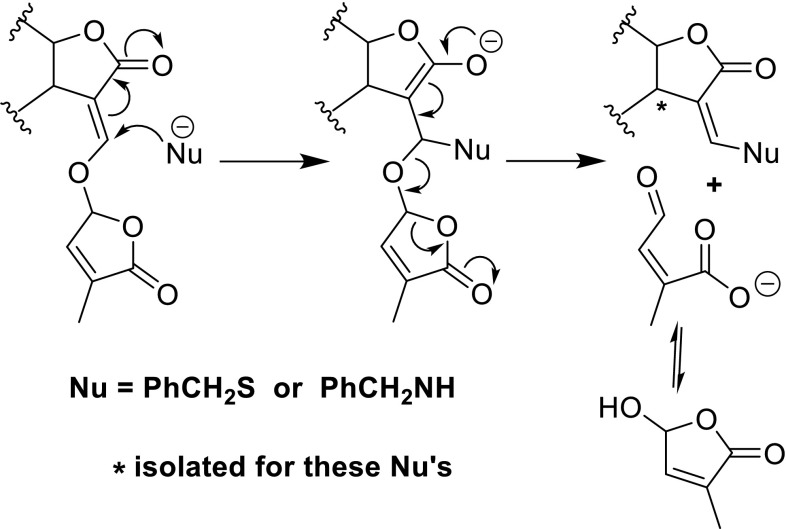
Tentative molecular mechanism for the mode of action via an addition–elimination reaction

The first step in the bioprocesses mediated by SLs involves the interaction of the SLs with a protein receptor. To shed light on this interaction knowledge of the protein structure is necessary. In the early days of SL research, when no protein structures were available it was tentatively suggested that a nucleophile at the receptor site, for instance an amino, thiol or hydroxyl group, would react with an SL by an addition–elimination reaction, resulting in a detachment of the D-ring (Fig. 11) (Mangnus and Zwanenburg 1992). Evidence for this suggested pathway was the isolation of ABC scaffold product derived from a reaction with benzylthiol and benzyl amine.

**Fig. 12:**
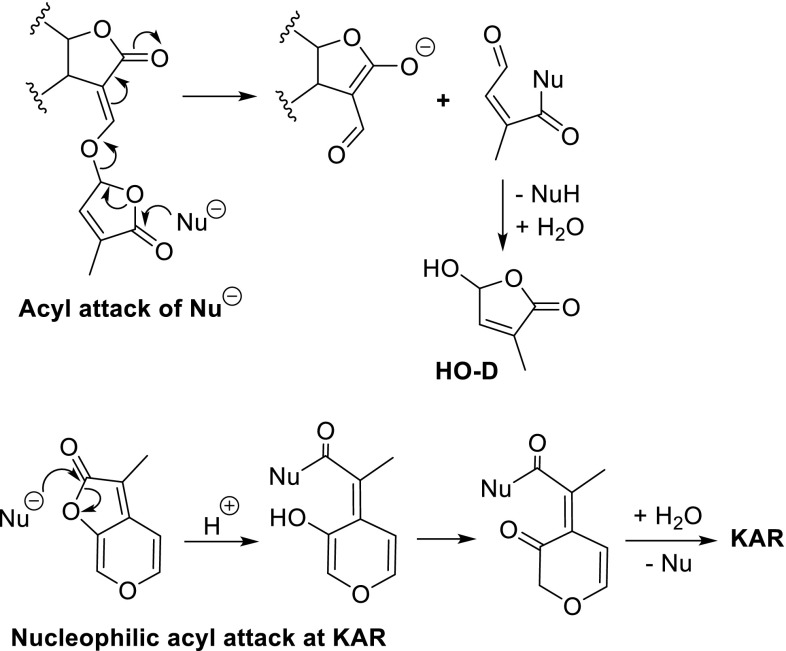
Tentative molecular mechanism for the mode of action involving a nucleophilic attack of ester carbonyl of the D-ring of an SL (acyl attack, *top line*). Idem for KAR (*bottom line*)

This mechanism was criticised by Scaffidi et al. (2012) in an attempt to unify the molecular mechanisms of KARs and SLs. Instead of an addition–elimination sequence, an acyl attack of the D-ring by a nucleophile was proposed as an alternative (Fig. 12). For KAR, a reclosure of the five-membered ring to KAR was proposed (Fig. 12). It should be noted, however, that an acyl attack of an ester is not in accordance with the generally accepted behaviour of esters, thus making this alternative mode of action for SLs less likely. Moreover, the isolation of the ABC-adduct (* in Fig. 11) obtained by the exposure of GR24 to either benzylthiol or benzylamine cannot be explained.

More recently, several studies of protein structures were reported which shed new light on the signal perception of SLs especially in SLs in shoot and branching inhibition (Hamiaux et al. 2012; Guo et al. 2013; Kagiyama et al. 2013; Nakamura et al. 2013; Zhao et al. 2013). The *DAD2* gene was identified from petunia which encodes for an α/β hydrolase protein DAD2 (Hamiaux et al. 2012). Similarly, rice genome *D14* encodes for the protein D14 (DWARF14) and a closely related homolog D14-LIKE (D14L) (Kagiyama et al. 2013; Zhao et al. 2013). The latter is also referred to as KARRIKIN INSENSITIVE 2 (KAI2) present in *Arabidopsis* and which is specific to karrikins (KARs) (Arite et al. 2009). The role of the *D14* gene products, whose sequence suggests that they belong to the α/β-fold hydrolase super family, received much attention because members of the α/β fold hydrolase superfamily are known to participate in hormone signalling for instance those involving gibberellin (GA) and the receptor GID1 (Ueguchi-Tanaka et al. 2005; Murase et al. 2008). The three protein structures, DAD2, D14 and KAI2 are almost superimposable, implying that they are orthologs.

The crystal structure of the protein DAD2 reveals an α/β hydrolase fold containing a canonical catalytic triad Ser-His-Asp with a large cone-shaped internal cavity capable of accommodating SLs (Hamiaux et al. 2012). The protein was incubated with racemic GR24 in a 1:20 ratio. After 18 h, no GR24 was left and formyl tricyclolactone (ABC=CHOH) resulting from the hydrolysis of GR24 was isolated by chromatography along with an unknown second product (probably an artifact) (Hamiaux et al. 2012). On the basis of this hydrolytic detachment of the D-ring it was proposed that SLs are essential for the signal transduction, in spite of the fact that the conditions for this hydrolysis experiment were far from biomimetic; similarly, incubation of D14 with GR24 resulting in hydrolysis products ABC=CHOH and hydroxy butenolide (D–OH) (Zhao et al. 2013; Nakamura et al. 2013). The latter most detailed study (Nakamura et al. 2013) revealed that the hydrolysis induced by D14 is stereospecific. (+)-GR24 underwent hydrolysis much faster than its antipode (−)-GR24. Differential scanning fluorimetry (DCF) measurements of DAD2 with increasing amounts of GR24 indicated a binding of GR24 with DAD2 in the ratio of 2:1. DCF measurements were also used to establish the interaction of SLs (GR24) with the protein D14 (Kagiyama et al. 2013).

Co-crystallisation of GR24 with D14 could not be accomplished. Zhao et al. (2013) observed in an attempted co-crystallisation experiment of rice D14 and GR24 an electron density that was assigned to 2,4,4-trihydroxy-3-methyl-3-butenal [(HO)_2_C=C(Me)-CH(OH)-CH=O] which was proposed as an intermediate en route to hydroxy butenolide (HO-D). Its formation was rationalised by an acyl transfer reaction (see Fig. 12, compared Scaffidi et al. 2012, mechanistically not generally accepted behaviour of esters) involving the D-ring and the serine unit of the catalytic triad to give the ring-opened product [SerCH_2_OC(=O)C(Me)=CHCH=O] which is then suggested to undergo a rotation around the olefinic bond (an energetically highly demanding conversion, unlikely to occur in the crystal lattice at ambient temperature) to give isomeric HO_2_CC(Me)=CH–CH=O. Subsequent addition of water to the –CH=C(Me)CO_2_H moiety gives the intermediate bound to Ser. Lactonization and elimination of water then results in HO-D. This sequence of events with two questionable steps lacks underpinning and is not an adequate explanation for the detachment of HO-D without further confirmation.

**Fig. 13:**
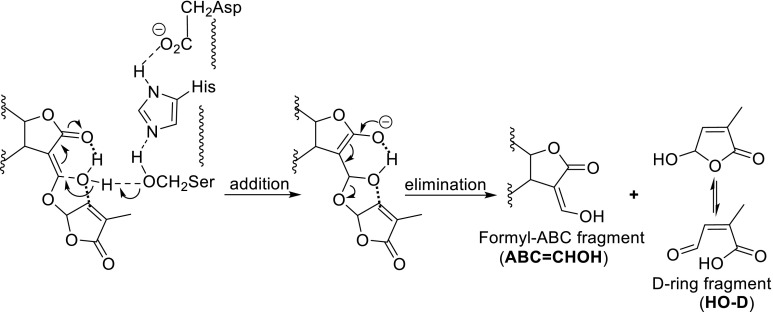
Proposal for the hydrolysis mechanism of SLs induced by the catalytic triad of Ser-His-Asp

A conceivable and more realistic mechanism for the detachment of the D-ring is shown in Fig. 13. Bidentate coordination of water fixes the rotation of the D-ring, which leads to a gain of entropy for the reactions to follow. Water is now favourably disposed for a vinologous water addition to the C-ring induced by the Ser unit of the catalytic triad. Subsequent elimination gives HO-D and the concurrent formation of the ABC=CHOH fragment. This detachment mechanism is in agreement with the one shown in Fig. 11 (Nu = H_2_O).

**Fig. 14:**
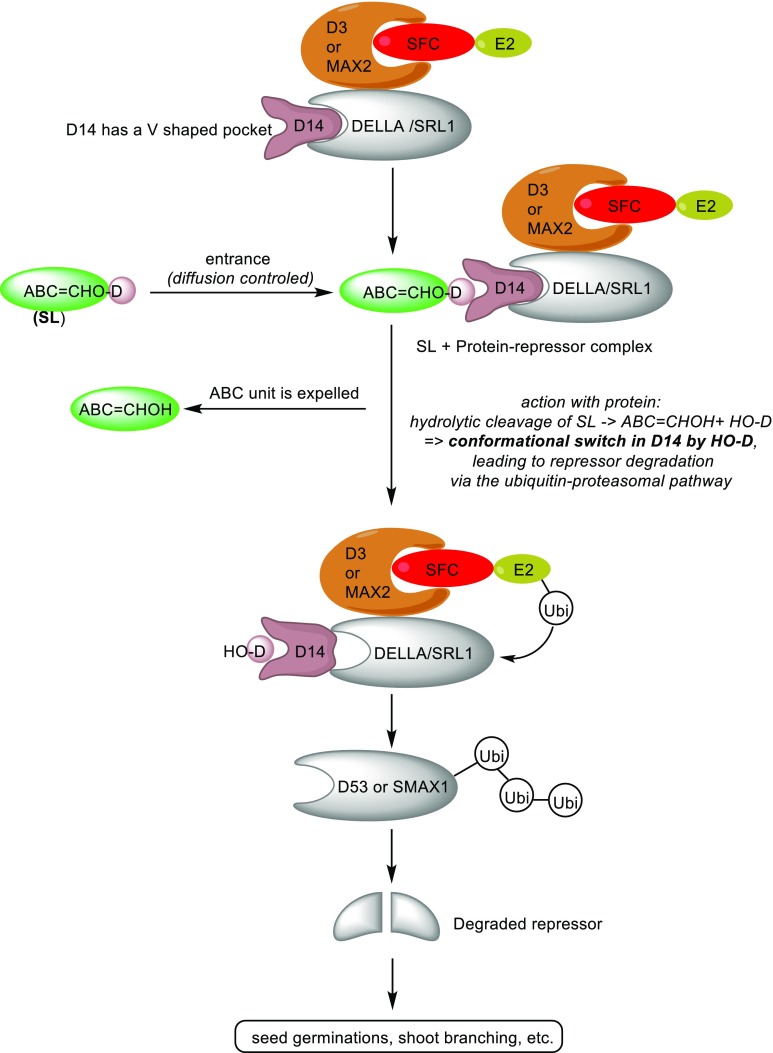
Schematic presentation of the interaction of an SL with the receptor protein D14

Also the SL signalling involves interaction with PhMAX2A to initiate an SCF-mediated signal transduction pathway, but the details were not unveiled by Hamiaux et al. (2012). The involvement of an α/β-hydrolase in the SL signal transduction is reminiscent of the GA reception system in which the GID1 receptor changes conformation upon hormone binding (Ueguchi-Tanaka and Matsuoka 2010). Therefore, the question arises whether D14 and the like exert their action in an analogous manner. The X-ray of D14 reveals that the helical lid which is typical for GID1, is not present in D14 (Kagiyama et al. 2013). Most revealing in this respect is the report by the Asami group (Nakamura et al. 2013; see also Jiang et al. 2013; Zhou et al. 2013; Koltai 2014; Seto and Yamaguchi 2014). It was convincingly shown that after the detachment of HO-D from the SLs by the catalytic action of the Asp-His-Ser triad that this hydroxy butenolide was accommodated in the active pocket. This induces a conformational change of the pocket to allow an interaction of D14 and the DELLA protein SLR1 resulting in a proteasomal degradation in a manner similar to GID1 in GA signalling (Hedden 2008; Murase et al. 2008) allowing the gene transcription to occur. The sequence of events is schematically shown in a cartoon (Fig. 14). Remarkably, introduction of HO-D as such did not induce the D14-SRL1 interaction. Probably, the ABC moiety serves as a lipophilic carrier for HO-D. It should be noted that the ABC=CHOH moiety after being detached from the D-ring will be expelled. The stereoselectivity for GR24 can readily be explained by the difference in diastereochemical interactions of (+)-GR24 and (−)-GR24 with the chiral walls of the V-shape cavity.

The interaction of the karrikins (KARs) with KAI2 protein was also clarified using an X-ray structure. Interestingly, it was found that the karrikin molecule is not hydrolyzed by the protein (Janssen and Snowden 2012; Guo et al. 2013). The KAR molecule is situated in the opening to the active site close to a helical domain but distal from the canonical catalytic triad of the α/β/hydrolase. Without undergoing any molecular change KAR is inducing a conformational change in the KAI2 protein which initiates the signal transduction production process in close analogy to the mode of action of gibberellins. It should be noted that this mode of action of KAR demonstrates that SLs and KARs are entirely different molecular entities, as already outlined in Fig. 10.

**Fig. 15:**
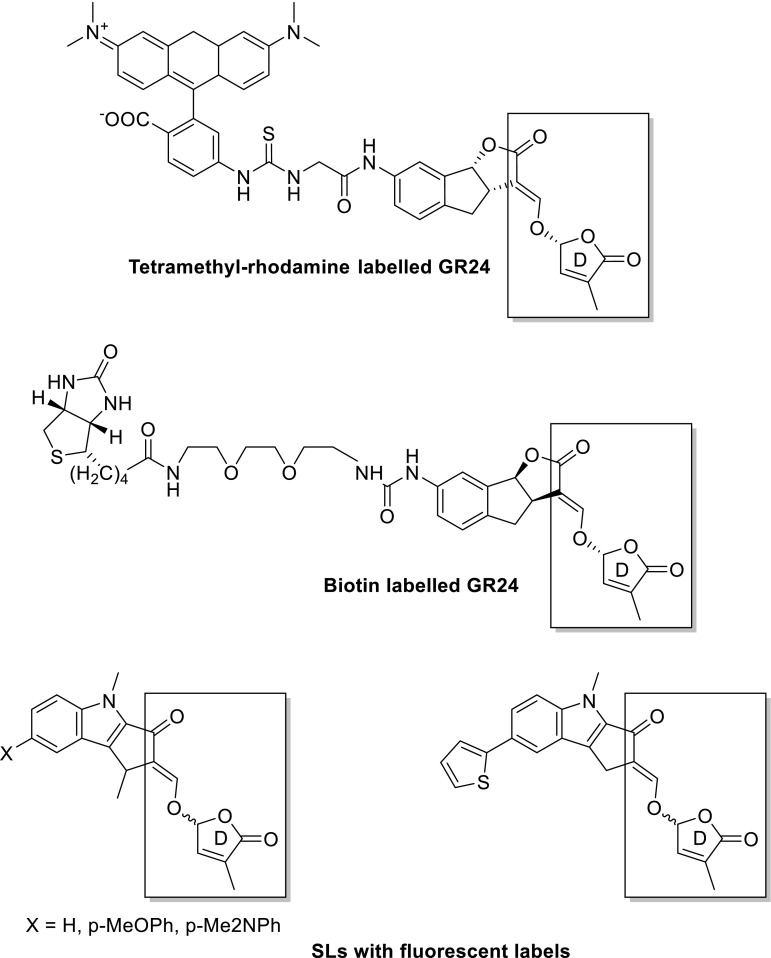
SLs with functional labels; the bioactiphore is in the *boxed* part

A receptor protein for the germination of seeds of parasitic weeds is not yet available and accordingly no mode of action on a molecular level can be given. Nevertheless, we speculate that the protein(s) involved may be (very) similar to D14. Support for this hypothesis is that the bioactiphore for germination, see Fig. 3, has the D-ring connected with the C-ring via an enol ether unit which allows the same SL-mediated mechanism with a crucial role for a canonical catalytic triad and a hydrolytic detachment of the D-ring, as shown in Fig. 13. It should be noted that in the model compound for germinating agents, see Fig. 3, there is a considerable freedom in choosing the substituent for the A-ring. Striking examples are the germinating agents with functional labels, see for typical examples Fig. 15 (Reizelman et al. 2003; Bhattacharya et al. 2009; Prandi et al. 2011; Rasmussen et al. 2013a, b).

All these compounds are remarkably active as germinating agents. After hydrolytic detachment of HO-D by interaction with the protein, ABC=CHOH fragment carrying the large substituent will undoubtedly be expelled from the active cavity. As a consequence, a fluorescent signal was measured upon interaction with a seed of a parasitic weed, may be due to the expelled fragment and not to the fluorescent SL in the receptor protein. In addition, protein fishing experiment may be frustrated by the enzymatic detachment of HO-D and the concurrent removal of the labelled ABC fragment from the protein. The ‘hook’ in the protein is detached from the ‘fishing line’ which is expelled.

The first attempts on the identification of a receptor protein of *S. hermonthioca* were reported recently (Toh et al. 2015). Using expression in *Arabidopsis,* it was shown that *ShHTLs* [*Striga HYPOSENSITIVE TO LIGHT/KARRIKIN INSENSITIVE 2* (*HTL/KAI2*); diverged family of α/β hydrolase-fold proteins related to D14] might be good candidates. However, isolation of a receptor protein from seeds of parasitic weeds has not yet been achieved.

**Fig. 16:**
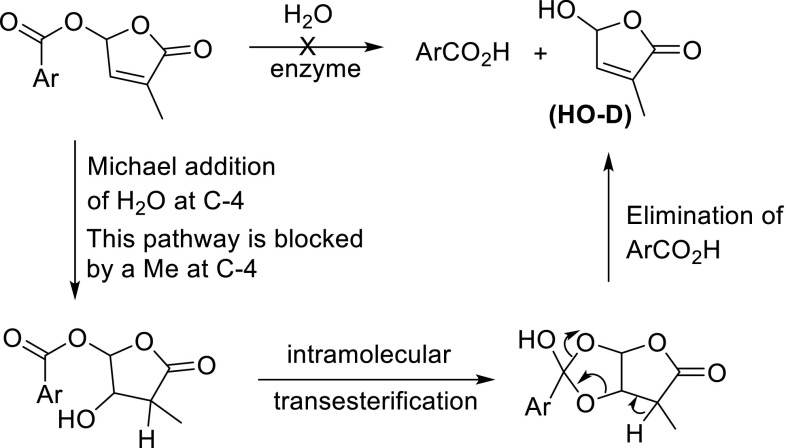
Tentative mode of action for SL mimics having an aroyloxy substituent at C-5 the D-ring

It remains to fit the activities of the SL mimics shown in Figs. 6 and 9 into the above patterns for the interactions with receptor proteins. Seemingly, the mode of action of the 5-aroyloxy-substituted butenolides (Fig. 6) can be readily rationalised by assuming a hydrolytic removal of the ester unit to give HO-D which then will induce a conformational change of the protein pocket in a manner described above for SLs. Nakamura et al. (2013) indeed suggested such a hydrolysis for the interaction of 3,5-dimethoxybenzoyloxy and anthracene-9-carbonyloxy butenolide with D14. These SL mimics inhibit the tiller bud outgrowth in rice. The benzoyloxy butenolides shown in Fig. 6 act as germination stimulants for parasitic weeds (Zwanenburg and Mwakaboko 2011; Zwanenburg et al. 2013). Assuming that the mode of action for bud outgrowth inhibition and germination of seeds of parasitic weed on the protein level take an analogous pathways for these aroyloxy SL mimics, then this suggested hydrolysis of the ester substituent cannot account for the blocking effect of a methyl group at C-4 of the butenolide ring on the germination activity (Zwanenburg et al. 2013). Hence, tentatively a detour mechanistic pathway is proposed involving an initial Michael addition of water to the butenolide, followed by an intramolecular transesterification to give the HO-D and the corresponding benzoic acid (Fig. 16). It is supposed that the Michael addition of water, which is essential for this pathway, is not possible when a methyl group is present at C-4. An earlier proposed mechanistic explanation (Zwanenburg and Pospisil 2013; Zwanenburg et al. 2013) is not correct as it does not lead to HO-D.

For the debranone type SLs (Fig. 6), a different mode of action must be operative. This pathway has to account for the observation that 3,4-dimethyl-5-*p*-chlorophenylthio-butenolide (see Fig. 9) is highly active in shoot branching control (Boyer et al. 2012, 2014). This implies that a Michael addition of water at C-4 of the D-ring cannot be part of the mode of action on the protein level.

**Fig. 17:**
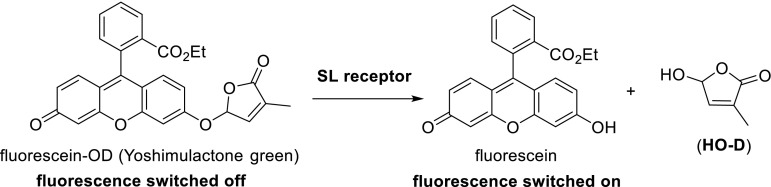
Fluorescein tagged SL mimics having a fluorescence turn-on probe

The aryloxy and arylthia substituent at C-5 of these SL mimics are connected with the D-ring via an acetal type unit. Accordingly, a hydrolytic detachment of the HO-D as shown for SLs cannot be envisaged. Very recently, highly relevant new information about SL mimics was reported by the Tsuchiya et al. (2015). They designed an SL mimic with a fluorescent turn-on probe based on fluorescein (Fig. 17). Conceptually, the design of this sophisticated SL mimic resembles the “prodrug approach” (Han and Burgess 2010): the D-ring tagged with fluorescein reaches the active site of the receptor protein whereupon the HO-D is released to start the signal transduction process. This SL mimic called Yoshimulactone green (YLG) stimulates germination of *S. hermonthica* seeds with simultaneous release of fluorophoric fluorescein. Practically, all germinated seeds emitted fluorescence. YLG is almost as active as the standard germination stimulant GR24. The hydrolysis products of YLG were isolated and analyzed by LS/MS. It was shown that YLG binds and acts via *ShHTLs*, the diverged family of α/β hydrolase-fold protein in *S.hermonthica.* This protein clearly must be capable of hydrolyzing an acetal unit. YLG also interacts with AtD14, the D14 homolog derived from Arabidopsis, again releasing fluorescein. This elaborate study of Tsuchiya et al. (2015) reveals that SL mimics are hydrolyzed by AtD14 and by the *ShHTL* receptor proteins in *Striga*. This hydrolysis is undoubtedly facilitated by the very good leaving ability of the fluorescent probe (Tsuchiya et al. 2015). The debranones shown in Fig. 6 will follow the same pattern as described for YGL, but the leaving ability of the aryloxy group is less pronounced implying that these debranones are less efficient stimulants (Tsuchiya et al. 2015).

Now it may be concluded that the mode of action of SLs, SL analogs and SL mimics show a consistent picture in all cases, the release of HO-D is the essential prime trigger for the cascade of reactions leading to the signal transduction.

## Applications of SLs

SLs, their analogs and mimics have a great potential for applications in agriculture. The control of parasitic weeds is under active investigation. One option for this is the suicidal germination approach. A germination stimulant, preferably a readily accessible synthetic analog, is applied to the field in the absence of a host. Seed of the weed will germinate but due to the lack of nutrients they die. After that the host plant, usually an important crop, can be planted which then does not suffer anymore from the parasitizing weed (Zwanenburg et al. 2009). Details will be described in a forthcoming review (Zwanenburg, accepted for publication in Pest  Manag Sci).

The recent finding that SLs play an essential role in the control of plant architecture led to extensive studies to improve the structure of agriculturally important plants. Details are, however, beyond the scope of this review.

## Conclusions and future outlook

The area of strigolactones is rapidly evolving. In recent years much new insight was obtained in the structure and bio-properties of naturally occurring SLs, but there is still much to gain. Reliable models have been developed for the design and synthesis of SL analogs with excellent bio-activity, but further fine tuning is necessary. The SL mimics constitute an important new group of simple compounds with a high bio-activity. Further development of SL mimics is highly relevant, also in connection with possible applications. Insight into the mode of action has been considerably improved. A consistent picture for SLs, SL analogs and SL mimics has been developed, but more information is needed to fully understand the interaction of SLs, its analogs and mimics with proteins. The role of SLs *in planta* for the control of plant architecture received much attention and will do so in the years to come. So far the protein receptors of seeds of parasitic weeds and AM fungi have not been isolated and identified; here lies an interesting challenge for the future. The molecular understanding of processes in which SLs play a dominant role is of utmost importance and may provide new leads for future research in this exciting area of plant hormones.

### *Author contribution statement*

BZ (senior author) 60 %, and both coauthors equal for the remaining part.

## Abbreviations

AM fungi: Arbuscular mycorrhizal fungi
KAR: Karrikins
SL: Strigolactone
